# Role of LIGHT in the Inflammatory Mechanisms of Psoriasis via Upregulation of Proliferation and Cytokine Production of Keratinocytes

**DOI:** 10.1002/iid3.70343

**Published:** 2026-02-12

**Authors:** Cheng‐bin Ye, Cheng‐wen Fei, Ting Cao, Xu‐yang Zhou, Ying Zou

**Affiliations:** ^1^ Allergic Dermatoses Clinical Center, Shanghai Skin Disease Hospital, School of Medicine Tongji University Shanghai China

**Keywords:** HaCaT cells, NF‐kappa B, psoriasis/immunology, signal transduction, TNFRSF14 protein—human, TNFSF14 protein—human

## Abstract

**Background:**

The pathogenesis of psoriasis is associated with abnormalities in immune pathways. HVEM is known as a receptor of LIGHT (homologous to lymphotoxins, inducible, and competes with HSV glycoprotein D), which is a newly identified member of the TNF superfamily. The expression of HVEM and LTBR (another LIGHT receptor) has been found to be increased in the skin of psoriasis patients. This indicates the potential role of LIGHT and its receptors in the pathogenesis of psoriasis. Therefore, the objective of this study was to examine the effect of LIGHT on keratinocyte proliferation and its therapeutic potential in the treatment of psoriasis.

**Methods:**

We used immunohistochemistry to examine their expression in psoriasis‐affected and normal tissue samples. We treated cells of the keratinocyte cell line HaCat with LIGHT protein, anti‐HVEM and anti‐LTβR antibodies, HVEM interference and LTβR interference RNA vectors, and NF‐κB and JNK/AP‐1 inhibitors at various concentrations and for various times, separately or simultaneously. The expression of NF‐κB was examined by immunofluorescence staining, and the expression of inflammatory proteins was measured with ELISA. Further, the viability of HaCat cells was examined with a CCK‐8 kit. In addition, flow cytometry was used to detect the expression of HVEM and LTBR on HaCat cells.

**Results:**

We found that LIGHT treatment of HaCat cells promoted the nuclear translocation of NF‐κB. Further, the expression of p‐c‐Jun, IL‐6, IL‐8, PGI2, and PTGS2 was increased in response to LIGHT treatment, but the expression of these factors was decreased when the LIGHT receptors were blocked or NF‐κB and JNK/AP‐1 expression was inhibited. We also found that the viability of HaCat cells was consistent with the expression of pro‐inflammatory factors.

**Conclusions:**

The present findings indicate that the JNK/AP‐1‐HVEM‐LIGHT pathway played a role in the viability of human keratinocytes and the expression of IL‐6, IL‐8, PGI2, and PTGS2. Thus, the JNK/AP‐1‐HVEM‐LIGHT pathway might be a potential target for the treatment of psoriasis.

## 1 Background

Psoriasis is a chronic, lifelong immune‐related disease for which the inherited factors remain largely unknown. This disease is characterized by inflammation of the skin and patches of red and flaky areas [1]. The prevalence of the condition is estimated to range from 1% to 5% in the worldwide population [2, 3, 4, 5]. One of the critical pathogenic mechanisms of psoriasis is thought to be abnormal immune regulation. Psoriasis is a complex disease associated with abnormalities in the growth and differentiation of skin cells [6, 7, 8, 9, 10].

The development of psoriasis is triggered by the activation of immune cells to produce tumor necrosis factor (TNF) and interleukin (IL)‐23 and the stimulation of Th17 autoimmune cells to migrate into the epidermis, where they are recognized by epidermal autoantigens and produce the cytokines IL‐17 and IL‐22; these molecular mechanisms are associated with hyperproliferation of keratinocytes and remodeling of the skin [6]. In particular, the TNF‐IL‐23‐Th17 inflammatory pathway appears to be crucial in the pathogenesis of chronic psoriasis. Understanding the pathogenesis of psoriasis would be a useful basis for the development of immune therapies that target pathogenic cytokines, including treatment with anti‐TNF, anti‐IL‐23, and anti‐IL‐17 antibodies [11, 12, 13]. Based on the evidence for the role of TNF‐α in the pathogenesis of psoriasis, one selective inhibitor (etanercept) and two antibodies (infliximab and adalimumab) of TNF‐α have been approved for the clinical treatment of psoriasis [14, 15]. Treatment with these biological agents has resulted in considerable improvement in a large number of patients with psoriasis; however, a considerably large number of patients still do not respond to these drugs [16]. Therefore, it is necessary to further investigate the molecular mechanism of psoriasis pathogenesis, which is greatly helpful to discover new biomarkers and therapeutic targets for psoriasis treatment.

Therefore, it is necessary to further investigate the molecular mechanisms underlying psoriasis pathogenesis in order to identify new biomarkers and therapeutic targets for psoriasis treatment.

Many members of the TNF superfamily are emerging as mediators and modulators of immune‐related disorders. Another family member expressed by T lymphocytes that is homologous to lymphotoxins, exhibits HVEM‐inducible expression, and competes with HSV glycoprotein D for HVEM, namely, LIGHT, has recently been identified. LIGHT is also known as tumor necrosis factor superfamily member 14 (TNFSF14) and is a newly identified secreted member of the TNF superfamily [17] that binds to its specific receptors HVEM and LTBR [18, 19]. LIGHT plays multiple roles in physiological and pathological processes in multiple tissues and organs [20, 21, 22, 23]. LIGHT and its receptor on cellular surfaces also play a key role in the pathogenesis of inflammatory‐response diseases [24, 25, 26]. In our previous study, we demonstrated that the expression of LIGHT and its receptor in tissue samples of psoriasis was higher than the expression in normal tissue samples. This implies that LIGHT and its receptor might be involved in a crosstalk with the classical TNF immune response pathway in the pathogenesis of psoriasis.

The aim of our study was to investigate the role of the LIGHT protein in autoimmune‐associated cytokine production pathways in psoriasis. In addition, the function of the downstream immune‐response effectors of LIGHT in human keratinocytes was determined using antibody and gene‐silencing methods.

## 2 Methods

### 2.1 Human Subjects

Five psoriatic skin samples were obtained by punch biopsy from patients with psoriasis, and basic demographic information, including age and gender, was collected. Non‐psoriasis normal skin samples were obtained from surgical discard specimens of healthy donors. The study was approved by the Ethical Committees of Shanghai Skin Disease Hospital (Shanghai, China) and was performed in accordance with the Declaration of Helsinki (Approved number: 2016‐012). Written informed consent was obtained from all participants.

### 2.2 Histological Assessment and Immunohistochemistry

The tissues were fixed with 4% paraformaldehyde and embedded in paraffin. Skin pathology was examined by H&E staining. For immunohistochemistry, after deparaffinization and rehydration, antigen retrieval was performed in EDTA buffer using a high‐pressure cooker. Tissue sections were incubated overnight at 4°C with primary antibodies against HVEM, LTβR, and LIGHT, followed by a 1‐h incubation at room temperature with a fluorescein‐labeled secondary antibody. Images were captured under a Leica fluorescence microscope (Leica Microsystems, Wetzlar, Germany).

### 2.3 Cells and Reagents

The human keratinocyte cell line HaCat was cultured in Dulbecco modified Eagle's medium (DMEM; Thermo Fisher Scientific Inc., Waltham, Massachusetts, the United States) with 10% fetal bovine serum and 1% penicillin–streptomycin at 37°C in a humidified environment with 5% CO_2_. PBS was acquired from Sigma‐Aldrich Inc. Other reagents, including trypsin, Lipofectamine 2000, primers, TRIzol, SuperScript III Reverse Transcriptase, SYBR Green I, DEPC H2O, Platinum Taq DNA polymerase, and 100 mM dNTPs, were obtained from Invitrogen. RNase inhibitor, endonucleases, EcoRI/BamHI, T4 DNA ligase, and GeneRuler DNA ladder were sourced from Fermentas. Gel extraction (AP‐GX‐50) and plasmid extraction kits (AP‐MN‐P‐50) were purchased from Corning Inc. *Escherichia coli* DH5α competent cells were purchased from Takara Biotechnology Inc.

### 2.4 FCM

Surface proteins HVEM and LTBR on HaCat cells were detected by flow cytometry (FCM). Cells were washed with PBS twice and incubated with trypsin at 37°C for 1 min. Following digestion with trypsin, the cell suspension was centrifuged at 400 *g* at room temperature for 5 min. The cell pellet was resuspended with PBS, and the centrifugation and resuspension steps were repeated two times. Cells were blocked with 2% bovine serum albumin (BSA; Sigma‐Aldrich Inc.) for 30 min at room temperature. Anti‐HVEM antibody (1:500; ab47677, Abcam Inc.) and anti‐LTBR antibody (1:500; Abcam Inc.) were added to a 100‐μL cell suspension and incubated at 4°C for 30 min. The cells were centrifuged at 400 *g* at room temperature for 5 min and resuspended in PBS three times. Secondary IgG antibody (1:400; goat anti‐rabbit IgG/fluorescein isothiocyanate [FITC] against HVEM and goat anti‐mouse IgG/FITC against LTBR) was added to the cell suspension and incubated at 4°C for 30 min. The cells were then washed with PBS three times, resuspended in 500 μL PBS, and analyzed by FCM. Data were acquired on an LSRII flow cytometer (BD Biosciences) and analyzed with the FlowJo software. Experiments were repeated three times.

### 2.5 Immunofluorescence Assay

HaCat cells were treated with 0.1 or 1 μg/mL LIGHT protein for 0, 30, or 60 min. Cells that had adhered to cover slips were fixed in 4% paraformaldehyde at room temperature for 10 min and blocked with 2% BSA (Sigma Inc.) for 30 min at room temperature. Cells were incubated with a primary antibody against NF‐κB p65 (1:500) at 4°C overnight. Following overnight incubation, the cover slips were washed with PBS and incubated in the dark with FITC‐conjugated goat anti‐rabbit secondary antibody (1:1000) at room temperature for 1 h. The cover slips were washed with PBS and stained with DAPI at room temperature for 5 min. Slides were prepared using an anti‐quenching mounting medium and observed under a fluorescence microscope (100×).

### 2.6 Cell Viability Assay

The viability of HaCat cells was measured using CCK‐8 (CK04; Dojindo Molecular Technologies Inc., Rockville, Maryland, the United States). CCK‐8 reagent was added to each well and incubated for 4 h. The absorbance was measured with a microplate reader at 490 nm. Experiments were repeated three times.

## 3 ELISA

HaCat cells were treated with 0.1 or 1 μg/mL LIGHT protein for 24 h, and the IL‐6, IL‐8, and PGI2 concentrations were detected by ELISA according to the manufacturer's instructions. Absorbance was measured using a microplate reader. Experiments were repeated three times.

### 3.1 Quantitative PCR

Total RNA was extracted using TRIzol reagent, following the manufacturer's protocol. A universal cDNA synthesis kit (Invitrogen Inc.) was utilized for reverse transcription. Each reaction solution contained 0.5 μL of random primers (0.2 μg/μL) and 1 μL SuperScript III reverse transcriptase (200 U/μL). The specific primers used are listed in Table 1. PCR was performed using an SYBR qPCR mix kit (Invitrogen Inc.) under the following conditions: pre‐denaturation at 95°C for 2 min, 40 cycles of denaturation at 95°C for 10 s, and annealing and polymerization at 60°C for 30 s and 70°C for 45 s. PCR was performed using a CFX96 Touch™ Real‐Time PCR Detection system (Bio‐Rad Inc.). Gene expression was determined and normalized to β‐actin expression [27]. The following rat β‐actin primers were used: forward, 5′‐AGGGAAATCGTGCGTGAC‐3′; reverse, 5′‐CGCTCATTGCCGATAGTG‐3′. The 2−ΔΔCq method was used to calculate the PCR results. The fluorophore used is included in the SYBR qPCR mix kit.

**Table 1:** Sequences of the primers used for qPCR.

Genes	Primers	Sequences (5′ to 3′)
IL6	Forward	CCCCTCAGCAATGTTGTTTGT
	Reverse	CTCCGGGACTGCTAACTGG
IL8	Forward	ACTGAGAGTGATTGAGAGTGGAC
	Reverse	AACCCTCTGCACCCAGTTTTC
PTGS2	Forward	aagatactcaggcagagatgatctaccc
	Reverse	ttaagcacatcgcatactctgttgtgt

### 3.2 Construction of Recombinant Plasmids and Lentiviral Packaging

According to the HVEM and LTBR sequences, three pairs of oligo sequences each were designed (Table 2). After annealing of the sequences, double‐stranded DNA was inserted into the lentivirus vector PDS019_pL_shRNA_F and amplified. The recombinant plasmids were extracted. The interference vectors (Table 3) and negative control were transfected into 293T cells for 48 h. qPCR was used to detect interference efficiency, and vectors with high interference efficiency were selected for virus packaging. Packaging mix (9 µg) and recombinant lentiviral plasmids (3 µg) were added to Opti‐Minimum Essential Medium (MEM, 1.5 mL; Invitrogen, Thermo Fisher Scientific Inc.) and mixed. Lipofectamine 2000 (36 µL) was mixed with Opti‐MEM (1.5 mL) and incubated at room temperature for 5 min. The plasmid solution and diluted Lipofectamine 2000 were then mixed and incubated at room temperature for 5 min. The mixture was added to a culture dish containing 293T cells, and the cells were cultured for 48 h. Cell supernatant was then collected, centrifuged at 1500 × g for 10 min at room temperature, and filtered. The virus solution was then condensed by centrifugation at 50,000 × g for 2 h at 4°C and re‐suspended in DMEM (Gibco, Thermo Fisher Scientific Inc.). Interference efficiency was measured by qPCR.

**Table 2:** Oligo sequences.

Gene target	Site/Mutation	Primers name	Direction	Oligo sequences (5′ to 3′)
HVEM	470	HVEM‐470	Forward	CACCGCAGTCCAGGTTATCGTGTGACGAATCACACGATAACCTGGACTGC
		HVEM‐470	Reverse	AAAAGCAGTCCAGGTTATCGTGTGATTCGTCACACGATAACCTGGACTGC
	924	HVEM‐924	Forward	CACCGAGCCTCGTCATCGTCATTGTCGAAACAATGACGATGACGAGGCTC
		HVEM‐924	Reverse	AAAAGAGCCTCGTCATCGTCATTGTTTCGACAATGACGATGACGAGGCTC
	947	HVEM‐947	Forward	CACCGCTCCACAGTTGGCCTAATCACGAATGATTAGGCCAACTGTGGAGC
		HVEM‐947	Reverse	AAAAGCTCCACAGTTGGCCTAATCATTCGTGATTAGGCCAACTGTGGAGC
LT‐βR	520	LT‐βR‐520	Forward	CACCGCACCTATGTCTCAGCTAAATCGAAATTTAGCTGAGACATAGGTGC
		LT‐βR‐520	Reverse	AAAAGCACCTATGTCTCAGCTAAATTTCGATTTAGCTGAGACATAGGTGC
	1402	LT‐βR‐1402	Forward	CACCGCAACATCTACATCTACAATGCGAACATTGTAGATGTAGATGTTGC
		LT‐βR‐1402	Reverse	AAAAGCAACATCTACATCTACAATGTTCGCATTGTAGATGTAGATGTTGC
	743	LT‐βR‐743	Forward	CACCGTACACACTGCGAGCTACTTTCCGAAGAAAGTAGCTCGCAGTGTGTA
		LT‐βR‐743	Reverse	AAAATACACACTGCGAGCTACTTTCTTCGGAAAGTAGCTCGCAGTGTGTAC

**Table 3:** Interference vectors.

Vectors	
CL791‐1	CL791‐1_PL‐shRNA‐GFP‐homo‐ HVEM‐470
CL791‐2	CL791‐2_PL‐shRNA‐GFP‐homo‐ HVEM‐924
CL791‐3	CL791‐3_PL‐shRNA‐GFP‐homo‐ HVEM‐947
CL792‐1	CL792‐1_PL‐shRNA‐GFP‐homo‐ LT‐βR‐520
CL792‐2	CL792‐2_PL‐shRNA‐GFP‐homo‐ LT‐βR‐1402
CL792‐3	CL792‐3_PL‐shRNA‐GFP‐homo‐ LT‐βR‐743

### 3.3 PPI Network Construction and KEGG Pathway Enrichment Analysis

The protein–protein interaction (PPI) network of differentially expressed proteins was constructed using the STRING database (version 12.0). The list of screened differentially expressed proteins was imported into STRING, with the organism set to “Homo sapiens” and an interaction score threshold of “high confidence” (> 0.7). The resulting interaction network was exported in TSV format. Subsequently, the network was visualized and analyzed using Cytoscape software (version 3.10.0). KEGG pathway enrichment analysis for proteins in the network was conducted based on the functional annotation provided by the STRING database. A false discovery rate (FDR) of < 0.05 was considered statistically significant for the enrichment results.

### 3.4 Statistical Analysis

Statistical data were analyzed with GraphPad Prism, version 5.0 (GraphPad Software Inc., La Jolla, California, the United States), and the results are presented as mean ± standard error. All experiments (including ELISA, cell viability assays, and flow cytometry) were performed with three independent biological replicates (*n* = 3). The Shapiro–Wilk test confirmed normality of all datasets. Comparisons between two groups used Student's *t*‐test; comparisons among three or more groups used one‐way ANOVA with Bonferroni correction for multiple comparisons. *p* < 0.05 was considered to indicate statistically significant differences.

## 4 Results

### 4.1 Increased Expression of LIGHT and HVEM in the Skin Tissues of Psoriasis Patients

**Figure 1:**
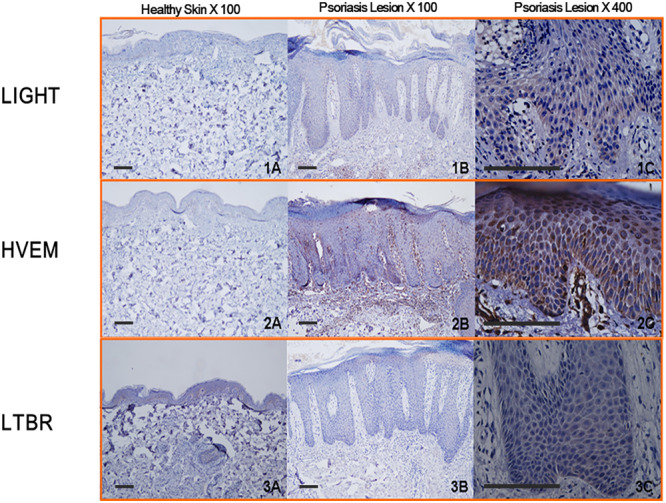
Comparison of LIGHT, HVEM, and LTBR expression in psoriasis‐affected and normal tissue samples. The skin samples were immunohistochemically stained for LIGHT, HVEM, and LTBR expression (original magnification, ×100 and ×400). Scale bar = 10 μm.

Our previous data had shown that LIGHT treatment can induce the pathological process of psoriasis under in vitro conditions, and they also indicated that LIGHT and its receptors might be involved in the pathological development of psoriasis. To verify this hypothesis, we collected tissue samples from psoriasis patients and normal skin samples and detected the expression of LIGHT and its receptors HVEM and LTBR by immunohistochemistry. Higher expression of LIGHT and HVEM, but not LTBR, was observed in the skin tissue of psoriasis patients (Figure 1).

### 4.2 Role of Increased Light Protein Expression in the Nuclear Translocation of NF‐κB and Significantly Increased Expression of p‐c‐Jun, IL‐6, IL‐8, PGI2, and PTGS2

**Figure 2:**
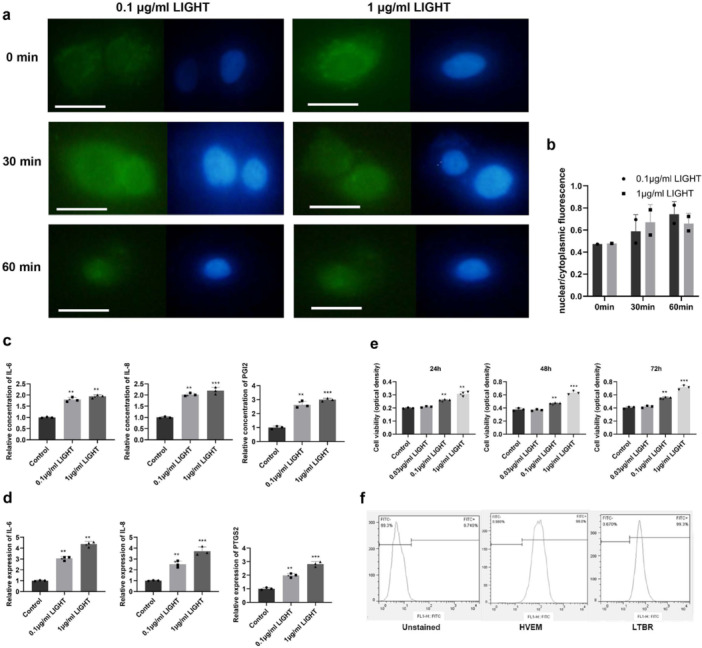
Effect of LIGHT on the expression of NF‐κB, pro‐inflammatory factors, and viability of HaCat cells. (a) Immunofluorescence images showing NF‐κB p65 (green) localization at the indicated time points after treatment with LIGHT (0.1 or 1 μg/mL). Nuclei were counterstained with DAPI (blue). Scale bar = 100 μm. (b) Nuclear/cytoplasmic fluorescence was analyzed by ImageJ. (c and d) Quantitative real‐time results showing levels of mRNA expression of various pro‐inflammatory factors. (e) Cell viability assessed by CCK‐8 assay after LIGHT treatment. The bars indicate the mean values in each group. **p < 0.05, ***p < 0.001. (f) Expression of HVEM and LTBR on the surface of HaCat cells was detected by FCM.

Human keratinocyte HaCat cells were treated with 0.1 and 1 μg/mL of recombinant LIGHT protein for 0, 30, and 60 min. Intracellular NF‐κB levels were detected by immunofluorescence analysis. LIGHT treatment was found to increase the nuclear localization of NF‐κB p65 (RelA), as the amount of NF‐κB p65 (RelA) was higher in the nucleus (stained in blue; Figure 2a) than in the cytoplasm (indicated by green staining). The effects of treatment with 0.1 and 1 μg/mL LIGHT protein on nuclear localization of NF‐κB were similar (Figure 2b).

HaCat cells were treated with 0.03, 0.1, and 1 μg/mL of LIGHT for 24, 48, and 72 h. The results show that treatment with 0.1 and 1 μg/mL LIGHT resulted in a marked increase in the viability of HaCat cells at 24, 48, and 72 h (*p* < 0.01, Figure 2e). Moreover, treatment of HaCat cells with 0.1 and 1 μg/mL of LIGHT for 24 h led to an increase in the expression of IL‐6, IL‐8, PGI2, and PTGS2 (*p* < 0.01, Figure 2c,d).

### 4.3 Significant Decrease in Cell Viability and Expression of IL‐6, IL‐8, PGI2, and PTGS2 Induced by Blockade of LIGHT Receptors

**Figure 3:**
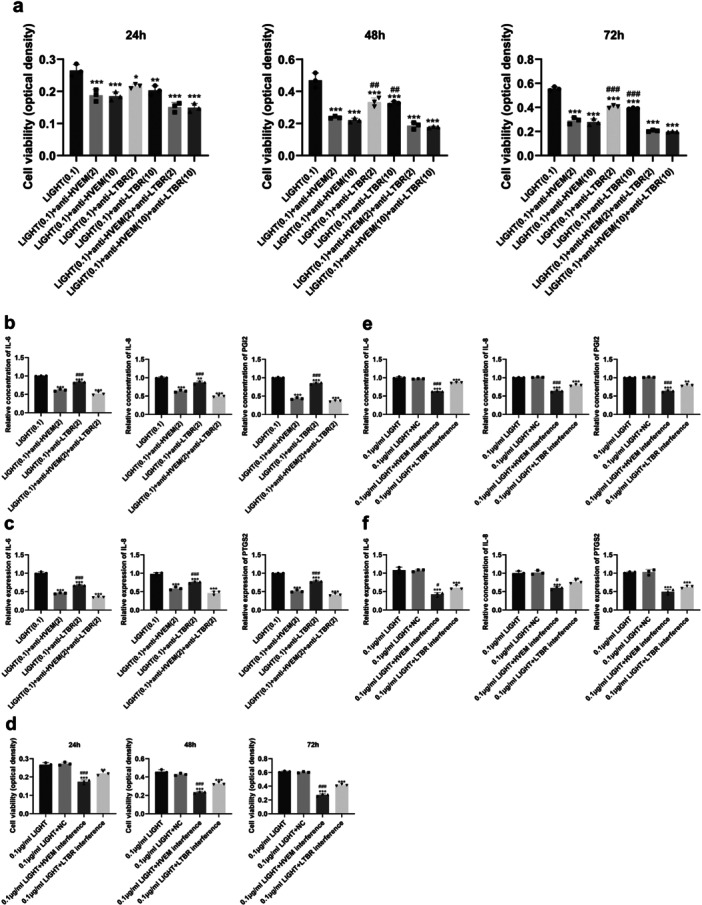
Effects of blocking HVEM and LTBR on the expression of IL‐6, IL‐8, PGI2, and PTGS2 and viability of HaCat cells. (a) Cell viability (CCK‐8 assay) upon LIGHT stimulation with or without receptor‐blocking antibodies. (b and c) qRT‐PCR analysis of inflammatory mediators after treatment with anti‐HVEM or anti‐LTBR antibodies. (d) Cell viability after LIGHT stimulation with HVEM or LTBR knockdown by siRNA. (e and f) qRT‐PCR analysis of inflammatory mediators following receptor knockdown. *p < 0.05, **p < 0.01, ***p < 0.001 versus the LIGHT‐treated group; ^#^ *p* < 0.05, ^##^ *p* < 0.01, ^###^ *p* < 0.001 versus the anti‐HVEM antibody‐treated group (a–c) or the HVEM interference RNA group (d–f).

Expression of HVEM and LTBR on the surface of HaCat cells was detected by FCM. Normal expression level of HVEM and LTBR was found on HaCat cells (Figure 2f). We used anti‐HVEM/anti‐LTBR antibodies to determine the effects of LIGHT treatment on human keratinocyte HaCat cells, which were divided into seven groups: (1) LIGHT (0.1 μg/mL) (the control group), (2) LIGHT (0.1 μg/mL) + anti‐HVEM antibody (2 μg/mL), (3) LIGHT (0.1 μg/mL) + anti‐HVEM antibody (10 μg/mL), (4) LIGHT (0.1 μg/mL) + anti‐LTBR antibody (2 μg/mL), (5) LIGHT (0.1 μg/mL) + anti‐LTBR antibody (10 μg/mL), (6) LIGHT (0.1 μg/mL) + anti‐HVEM antibody (2 μg/mL) + anti‐LTBR antibody (2 μg/mL), and (7) LIGHT (0.1 μg/mL) + anti‐HVEM antibody (10 μg/mL) + anti‐LTBR antibody (10 μg/mL). The viability of HaCat cells was assessed with Cell Counting Kit‐8 (CCK‐8). Compared to the control group, the cell viability of HaCat cells treated with anti‐HVEM and anti‐LTBR antibodies had significantly decreased at 24, 48, and 72 h (*p* < 0.05, Figure 3a). HaCat cells treated with the anti‐LTBR antibody had markedly higher cell viability at 48 and 72 h, as compared to HaCat cells treated with the anti‐HVEM antibody (*p* < 0.01, Figure 3a). In addition, HaCat cells were treated with 0.1 μg/mL LIGHT, 0.1 μg/mL LIGHT + 2 μg/mL HVEM, 0.1 μg/mL LIGHT + 2 μg/mL LTBR, and 0.1 μg/mL LIGHT + 2 μg/mL HVEM + 2 μg/mL LTBR for 24 h. With regard to the concentration of IL‐6, IL‐8, and PGI2 in the supernatant, the expression of IL‐6, IL‐8, PGI2, and PTGS2 in HaCat cells treated with anti‐HVEM and anti‐LTBR antibodies had significantly decreased compared to the control group at 24 h (*p* < 0.01, Figure 3b). Further, HaCat cells treated with the anti‐LTBR antibody exhibited dramatically higher expression of IL‐6, IL‐8, PGI2, and PTGS2 at 24 h, as compared to HaCat cells treated with the anti‐HVEM antibody (*p* < 0.001, Figure 3c).

We suppressed the function of HVEM and LTBR in HaCat cells by RNA interference with viral vectors. The interference efficiency of the vectors listed in Table 3 was detected by qPCR. The interference efficiency was 73%, 44%, 55%, 20%, 70%, and 56% for CL791‐1, CL791‐2, CL791‐3, CL792‐1, CL792‐2, and CL792‐3, respectively. The CL791‐1 (CL791‐1_PL‐shRNA‐GFP‐homo‐HVEM‐470) and CL792‐2 (CL792‐2_PL‐shRNA‐GFP‐homo‐LT‐βR‐1402) vectors were screened, and lentiviruses (LV473‐1_PL‐shRNA‐GFP‐homo‐HVEM‐470 and LV473‐2_PL‐shRNA‐GFP‐homo‐LT‐βR‐1402) were packed into vectors. HaCat cells were transfected with LV473‐1 and LV473‐2 for 48 h, and the interference efficiency was detected by qPCR. The interference efficiency of LV473‐1 against HVEM was 86%, and the interference efficiency of LV473‐2 against LTBR was 71%. HaCat cells were divided into four groups: (1) LIGHT (0.1 μg/mL) (the control group), (2) LIGHT (0.1 μg/mL) + negative control vector, (3) LIGHT (0.1 μg/mL) + HVEM interference by LV473‐1, and (4) LIGHT (0.1 μg/mL) + LTBR interference by LV473‐2. The cell viability of HaCat cells in which HVEM and LTBR expression was suppressed had markedly decreased at 24, 48, and 72 h compared to the control group (*p* < 0.01, Figure 3d). HaCat cells with HVEM suppression had markedly lower cell viability at 24, 48, and 72 h, as compared to HaCat cells in which LTBR expression was suppressed (*p* < 0.01, Figure 3d). Further, the expression of IL‐6, IL‐8, PGI2, and PTGS2 in HaCat cells with suppression of HVEM and LTBR expression had significantly decreased at 24 h, as compared to the control group (*p* < 0.01, Figure 3e). HaCat cells with suppression of HVEM expression exhibited markedly lower expression of IL‐6, IL‐8, and PGI2 at 24 h, as compared to HaCat cells with suppressed expression of LTBR (*p* < 0.05, Figure 3f).

### 4.4 Significant Decrease in Cell Viability and Expression of IL‐6, IL‐8, PGI2, and PTGS2 Induced by Inhibition of NF‐κB and JNK/AP‐1 Expression

**Figure 4:**
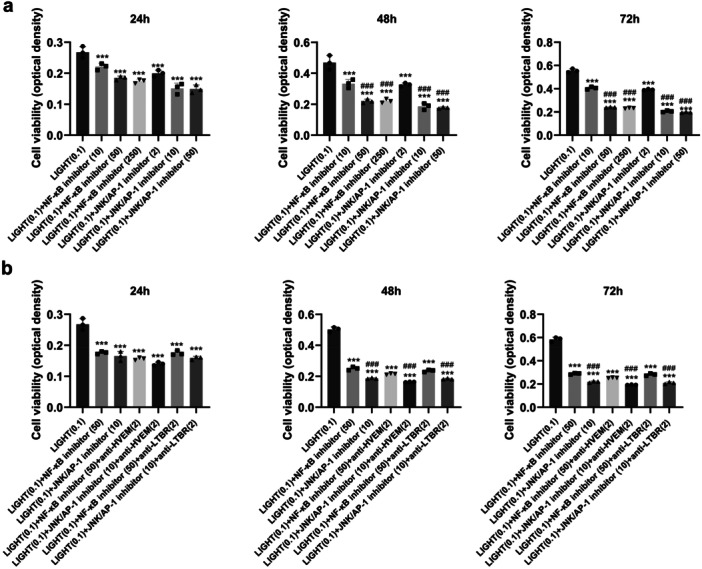
Effect of treatment with inhibitors of NF‐κB and JNK/AP‐1 on the viability of HaCat cells. Optical density was used as a measure of the cell viability of HaCat cells exposed to inhibitors of NF‐κB (a) and JNK/AP‐1 (b) for 24, 48, or 72 h. The bars indicate the mean values in each group. ***p < 0.001 versus the LIGHT‐treated group. ### *p* < 0.001 versus the former group.

**Figure 5:**
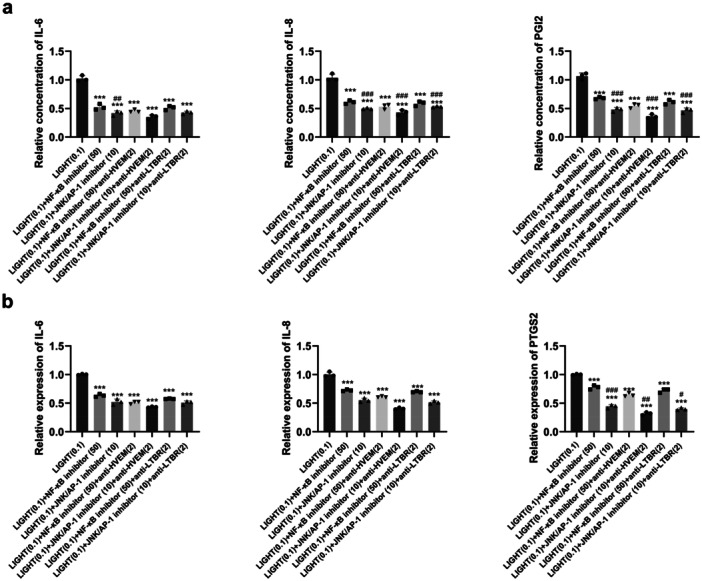
Effect of treatment with inhibitors of NF‐κB and JNK/AP‐1 on the viability and IL‐6, IL‐8, PGI2, and PTGS2 expression of HaCat cells. The viability (a) and expression of IL‐6, IL‐8, PGI2, and PTGS2 (b) of HaCat cells treated with different concentrations of NF‐κB and JNK/AP‐1 for 24 h are shown. ***p < 0.001 versus the LIGHT‐treated group. # *p* < 0.05, ##*p* < 0.01, ### *p* < 0.001 versus the former group.

HaCat cells were divided into seven groups: (1) LIGHT (0.1 μg/mL) (the control group), (2) LIGHT (0.1 μg/mL) + NF‐κB inhibitor (10 μM), (3) LIGHT (0.1 μg/mL) + NF‐κB inhibitor (50 μM), (4) LIGHT (0.1 μg/mL) + NF‐κB inhibitor (250 μM), (5) LIGHT (0.1 μg/mL) + JNK/AP‐1 inhibitor (2 μM), (6) LIGHT (0.1 μg/mL) + JNK/AP‐1 inhibitor (10 μM), and (7) LIGHT (0.1 μg/mL) + JNK/AP‐1 inhibitor (50 μM). Compared to Group 1, HaCat cells treated with inhibitors of NF‐κB and JNK/AP‐1 had markedly decreased cell viability at 24, 48, and 72 h (*p* < 0.001, Figure 4a). HaCat cells treated with 50 and 250 μM of the NF‐κB inhibitor had markedly decreased cell viability at 48 and 72 h compared to HaCat cells treated with 10 μM of the NF‐κB inhibitor (*p* < 0.001, Figure 4b). Further, HaCat cells treated with 10 and 50 μM of the JNK/AP‐1 inhibitor had markedly decreased cell viability at 48 and 72 h compared to HaCat cells treated with 2 μM of the JNK/AP‐1 inhibitor (*p* < 0.001, Figure 5). Therefore, 50 μM of the NF‐κB inhibitor and 10 μM of the JNK/AP‐1 inhibitor were used in the following experiments.

HaCat cells were divided into seven groups: (1) LIGHT (0.1 μg/mL) (the control group), (2) LIGHT (0.1 μg/mL) + NF‐κB inhibitor (50 μM), (3) LIGHT (0.1 μg/mL) + JNK/AP‐1 inhibitor (10 μM), (4) LIGHT (0.1 μg/mL) + NF‐κB inhibitor (50 μM) + anti‐HVEM antibody (2 μg/mL), (5) LIGHT (0.1 μg/mL) + JNK/AP‐1 inhibitor (10 μM) + anti‐HVEM antibody (2 μg/mL), (6) LIGHT (0.1 μg/mL) + NF‐κB inhibitor (50 μM) + anti‐LTBR antibody (2 μg/mL), and (7) LIGHT (0.1 μg/mL) + JNK/AP‐1 inhibitor (10 μM) + anti‐LTBR antibody (2 μg/mL). HaCat cells treated with the NF‐κB and JNK/AP‐1 inhibitors exhibited dramatically decreased cell viability at 24, 48, and 72 h compared to the control group (*p* < 0.001, Figure 5a). HaCat cells treated with the JNK/AP‐1 inhibitor had markedly decreased cell viability at 48 and 72 h compared to HaCat cells treated with the NF‐κB inhibitor (*p* < 0.001, Figure 5a). Further, HaCat cells treated with the NF‐κB and JNK/AP‐1 inhibitors had significantly decreased expression of IL‐6, IL‐8, PGI2, and PTGS2 at 24 h (*p* < 0.001, Figure 5b), and HaCat cells treated with the JNK/AP‐1 inhibitor had markedly decreased expression of PGI2 and PTGS2 at 24 h as compared to HaCat cells treated with the NF‐κB inhibitor (*p* < 0.05, Figure 5b).

### 4.5 Functional Integration of LIGHT/HVEM and IL‐17/IL‐23 Signaling Pathways via Shared Nodes in the PPI Network

**Figure 6:**
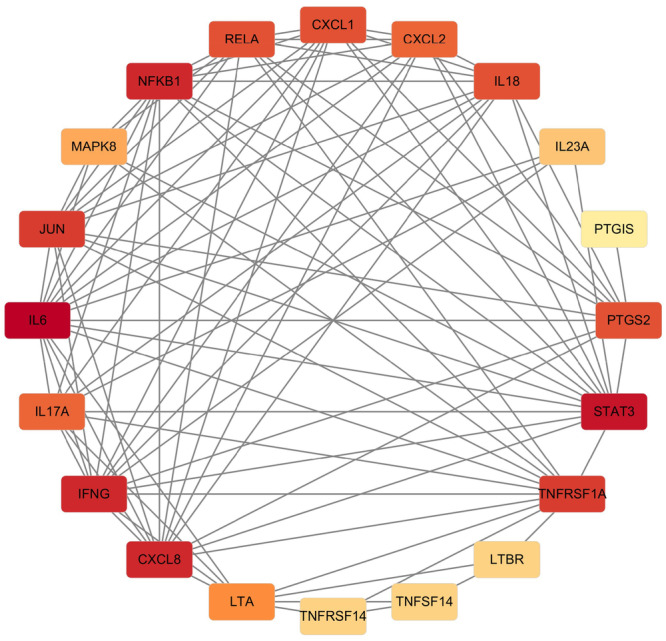
KEGG pathway enrichment analysis of differentially expressed genes. Enrichment significance is represented by color intensity (−log10(FDR)), and gene count per pathway is indicated by circle size.

**Figure 7:**
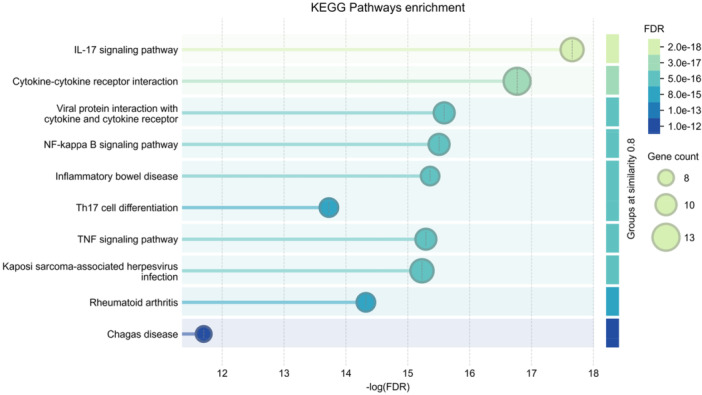
Protein–protein interaction network of psoriasis‐related signaling pathways. Nodes represent key proteins (e.g., TNFSF14/LIGHT, TNFRSF14/HVEM, IL‐17A, and IL‐23A), and edges indicate functional associations.

The PPI network depicts the close functional associations among the analyzed proteins. Key molecules such as IL6, IL17A, IL23A, IFNG, CXCL1, CXCL2, LIGHT (TNFSF14), HVEM (TNFRSF14), JUN, and RELA (a component of NF‐κB) are central nodes with dense connections. This high interconnectivity demonstrates a strong functional relationship between the LIGHT/HVEM pathway, IL23/IL‐17 signaling, and the NF‐κB transcription factor network (Figure 6). The enrichment analysis identified the top 20 significantly enriched KEGG pathways. The horizontal bars represent the enrichment significance (−log10(FDR)), with color intensity and length corresponding to increasing statistical significance. Key enriched pathways include those related to cytokine–cytokine receptor interaction, IL‐17 signaling pathway, chemokine signaling pathway, and NF‐κB signaling pathway (Figure 7).

## 5 Discussion

Our study provided evidence that LIGHT increased the viability and cytokine production of human keratinocytes. As keratinocytes predominated in the pathogenesis of psoriasis, together with other immunocytes, our findings also revealed potential involvement of LIGHT/HVEM signaling in the development of psoriasis. Psoriasis is characterized by over‐proliferating keratinocytes; our study suggests that LIGHT/HVEM signaling, which is upregulated in psoriasis, significantly promotes the proliferation and cytokine production of keratinocytes. We also found that blockade of LIGHT/HVEM by NF‐κB or JNK/AP‐1 inhibitor could diminish the effects of LIGHT on keratinocytes; therefore, targeting LIGHT/HVEM might help to suppress the over‐proliferation of keratinocytes in psoriasis.

HVEM and LTβR are two surface receptors of LIGHT on keratinocytes. In accordance, our study confirmed that blocking both HVEM and LTβR could diminish the effects of LIGHT on keratinocytes. We also found that blockade of HVEM inhibited the viability and cytokine production of keratinocytes to a greater extent.

Limited evidence exists regarding the effects of the LIGHT protein on human keratinocytes and its role in psoriasis. However, it is known that keratinocyte proliferation and deregulated cytokine expression are central in the pathogenesis of psoriasis. The current study provides evidence that LIGHT stimulates cellular viability, suggesting increased proliferation and production of cytokines and inflammatory mediators in human keratinocytes. The findings suggest that LIGHT may have a key role in the pathogenesis of psoriasis. Treatment with recombinant LIGHT protein increased the viability of human keratinocytes and upregulated the LIGHT‐induced expression of IL‐6, IL‐8, PGI2, and PTGS2. In addition, recombinant LIGHT treatment promoted downstream transcription of NF‐kB. Blockade of HVEM and LTBR attenuated LIGHT‐induced cellular viability and the expression of cytokines and inflammatory mediators in human keratinocytes. Our data suggest that LIGHT may be useful as a marker for psoriasis and that blocking LIGHT activity could be effective for the treatment of psoriasis.

Keratinocytes are one of the predominant cell types in the outer layer of the skin. In fact, an imbalance in skin homeostasis caused by aberrant proliferation of keratinocytes is involved in the development of chronic inflammatory skin diseases, including psoriasis. Keratinocytes, as cells of the innate immune system in the skin, can release a cluster of inflammatory molecules, which lead to aggravation of inflammatory skin diseases. In the present study, treatment with recombinant LIGHT protein was found to promote keratinocyte viability, suggesting enhanced proliferative activity, and this indicated the role of LIGHT in psoriasis. Blockade of the LIGHT receptor HVEM and its downstream molecules JNK/AP‐1 resulted in suppression of keratinocyte proliferation, and this also implies that the LIGHT pathway might potentially be involved in the pathogenesis of psoriasis.

NF‐kB is expressed in most cell types, including epithelial cells, and its nuclear translocation is known to induce the production of cytokines and promote inflammation [26, 28]. In addition, phosphorylated c‐Jun plays a role in the inflammatory response to stress stimuli by cytokines [29, 30, 31, 32, 33]. In the present study, LIGHT treatment caused nuclear localization of NF‐kB and expression of phosphorylated c‐Jun in human keratinocytes. These data indicate that, similar to the TNF‐α pathway, the LIGHT pathway in keratinocytes is also involved in the inflammatory signaling pathways of psoriasis.

IL‐6 and IL‐8, which act as both pro‐inflammatory and anti‐inflammatory cytokines, are produced by T cells, macrophages, and other cells, including epithelial cells, and stimulate immune response and induce chemotaxis and phagocytosis, especially at sites of inflammation during infection [34, 35]. PGI2, which is an effective vasodilator, and its precursor PTGS2 are prostaglandin members of the eicosanoid family that inhibit platelet activation, prevent platelet plug formation, and are induced during inflammation. PTGS2 is involved in the conversion of arachidonic acid to the precursor of PGI2 [36]. In the present study, recombinant LIGHT protein not only dramatically increased IL‐6, IL‐8, PGI2, and PTGS2 expression in human keratinocytes but also promoted the keratinocyte viability, suggesting enhanced proliferative activity. Based on the data, it can be concluded that LIGHT plays a role in the inflammatory responses of human keratinocytes and, therefore, in the pathogenesis of psoriasis.

HVEM, as a specific cell surface receptor of LIGHT, can activate both NF‐kb and JNK/AP‐1 [26, 37, 38, 39]. LTBR is the surface receptor of LTB and LIGHT, both of which are members of the TNF superfamily. HVEM and LTBR can both be detected on the surface of keratinocytes. In the present study, human keratinocytes treated with HVEM‐ and LTBR‐targeting antibodies or interfering RNA delivered via viral vectors exhibited significantly decreased cell viability, as well as decreased expression of IL‐6, IL‐8, PGI2, and PTGS2. Although blockade of the cellular surface receptors HVEM and LTBR had similar effects on inflammatory responses in human keratinocytes, HVEM (as the specific receptor of LIGHT) had higher efficiency than LTBR. This differential efficacy may be attributed to their distinct structural features, tissue‐specific distribution, or downstream signaling potency. The above results suggest that LIGHT and its specific receptor HVEM may play a potential role in the pathogenesis of psoriasis, and further investigation into their differential mechanisms may provide new insights for targeted therapy.

JNK is a downstream effector of LIGHT/HVEM, and the JNK signaling pathway regulates a number of biological processes through the activation of the transcription factor AP‐1 [40, 41, 42]. Apart from the critical role of AP‐1 in skin homeostasis [43, 44], induction of JNK/AP‐1/c‐Jun activity is known to lead to tumor progression and the development of malignant melanoma of the skin [45, 46]. In the present study, treatment with inhibitors of NF‐kb and JNK/AP‐1 resulted in a significant decrease in cell viability and a significant decrease in the expression of IL‐6, IL‐8, PGI2, and PTGS2. Moreover, JNK/AP‐1 might be a specific downstream effector of LIGHT/HVEM in human keratinocytes and has a higher efficiency than NF‐kb. The above results indicate that JNK/AP‐1, and not NF‐kb, is involved in the inflammatory responses of psoriasis. They also imply that the pathogenic mechanisms of psoriasis may, at least, partly be attributable to abnormal LIGHT/HVEM interaction and regulation of JNK/AP‐1.

It is known that TNF‐α is present at elevated levels in psoriasis and has multiple mechanisms of action that could account for the symptoms of psoriasis [47, 48]. TNF‐α has emerged as a target for the clinical treatment of psoriasis and is a crucial pleiotropic inflammatory cytokine that is involved in immune‐response regulation by inflammation [49]. In addition, TNF‐α has also been found to promote epidermal cell proliferation [50]. Further, the activation of TNF‐α transmembrane receptors on the cell surface induces a multitude of intracellular signaling pathways, including upregulation of the expression and nuclear translocation of NF‐κB, which can induce a cascade of events that include the production of inflammatory cytokines and chemokines such as IL‐1, IL‐8, and IL‐6, whose elevated levels are a pathological hallmark of psoriasis. The aforementioned immune responses indicate that TNF‐α plays an important pathogenic role in psoriasis. This is supported by the clinical remission of psoriasis symptoms observed in response to TNF antagonists that block its pro‐inflammatory effects [51]. However, it is still unclear why the effects of TNF‐α‐targeting drugs differ across patients. A significant proportion of patients do not respond to anti‐TNF‐α treatment or develop serious side effects. Adverse drug events in patients with psoriasis are mainly caused by the suppressive effects of the drugs on immune response, as this increases the risk of new or existing infection, fatal blood disorders, and tumors [52]. Therefore, it is important to investigate new biomarkers, such as TNF gene polymorphisms, that can help to predict the drug response of patients and develop new therapeutic targets.

Many members of the TNF superfamily and its receptor superfamilies, including LIGHT and HVEM, play a crucial role in the regulation of abnormalities in immune‐mediated disorders [23, 53, 54, 55]. LIGHT and its receptor HVEM are involved in the induction of JNK/AP‐1 and the expression of several autoimmune‐related inflammation cytokines and modulatory molecules. In the present study, we have demonstrated that the LIGHT/HVEM/JNK/AP‐1 pathway plays a major role in immune‐mediated skin inflammation and keratinocyte viability, suggesting enhanced proliferative activity. Specifically, it has been postulated and proven that the efficacy of neutralizing the ligand/receptor interaction of LIGHT/HVEM or inhibiting HVEM‐ or LIGHT‐induced transcription of JNK/AP‐1 in dampening keratinocyte activity suppresses psoriasis‐like symptoms under in vitro conditions. Further, suppression of the LIGHT pathway ameliorated the inflammatory activity of skin cells that is characteristic of psoriasis.

The findings of the present study imply a new possible mechanism of pathogenesis in psoriasis that involves the LIGHT pathway. However, LIGHT alone may not promote the psoriasis phenotypes; instead, it may promote the expression of common immune response proteins associated with psoriasis in human keratinocytes. The results of our experiments involving blockade of the LIGHT receptor HVEM (using an anti‐HVEM antibody or an HVEM‐silencing virus) and/or their downstream effector (using a JNK/AP‐1 inhibitor) suggest new potential targets for psoriasis patients who do not respond to anti‐TNF‐α treatment. However, further studies are required to investigate how the LIGHT pathway interacts with the disease‐specific cytokines IL‐23 and IL‐17 and how these pathogenic mechanisms differ from those of the TNF‐α pathway.

Our integrated analysis suggests significant crosstalk between the LIGHT/HVEM and IL‐23/IL‐17 signaling pathways. The PPI network reveals that proteins from both pathways are highly interconnected and converge on central pro‐inflammatory transcription factors, particularly NF‐κB (NFKB1, RELA) and AP‐1 (JUN). The co‐enrichment of these pathways and their physical connectivity indicate their signals are integrated to sustain a robust inflammatory response. This synergy is evident in the shared upregulation of key downstream effector molecules, such as the chemokines CXCL1, CXCL2, and CXCL8/IL8 and cytokines like IL‐6 and IL‐18. Furthermore, a potential feed‐forward loop is suggested: the inflammatory milieu fostered by LIGHT/HVEM signaling (e.g., IL‐6 production) can promote Th17 cell differentiation and sustain the IL‐17/IL‐23‐driven response, potentially leading to a chronic inflammatory state. In conclusion, the crosstalk between these pathways, centered on shared hubs like NF‐κB, synergistically drives a core inflammatory gene program, underpinning the pathogenic process. Animal studies by Sheng‐jie Long et al. demonstrated that the TNFSF14‐HVEM/LTβR pathway upregulates the expression of IL‐23/IL‐17 axis‐related inflammatory cytokines, thereby revealing a synergistic effect in inflammation amplification [56].

It has been reported that the TNF‐like ligand 1A (TL1A) is a key modulator of immune response and is critically involved in the pathogenesis of several autoimmune diseases, including psoriasis [56, 57]. TL1A binds to its two cognate receptors, both of which are members of the TNF receptor superfamily [58]. The expression of TL1A and its two receptors was also reported to be significantly increased in skin lesions, serum, and peripheral blood mononuclear cells from patients with psoriasis. Moreover, TWEAK (TNFSF12), a member of the TNF superfamily, is thought to critically contribute to the dysregulated expression of cytokines/chemokines and skin inflammation in psoriasis [59, 60, 61]. All these studies about the role of TL1A, TWEAK, and LIGHT in psoriasis speculated that not only TNF‐α but also other members of the superfamily are central mediators of inflammatory regulation in autoimmune diseases and could be involved in the pathogenesis of psoriasis. To date, the comparative roles and potential interactions among these pathways have not been thoroughly investigated.

The activation and nuclear translocation of NF‐κB by TNF‐α stimulate a major pathogenic immune response in psoriasis [62]. Herein, we determined that JNK/AP‐1 inhibition was more effective than NF‐kb inhibition in suppressing the dysregulation of immune responses induced by LIGHT in a keratinocyte cell model of psoriasis. This implies that JNK/AP‐1, and not NF‐kb, plays a pivotal role in the LIGHT‐related pathogenic mechanisms of psoriasis. Thus, JNK/AP‐1 might be a promising therapeutic target for the treatment of some subtypes of psoriasis. Recent studies have also reported that AP‐1 is involved in the initiation of skin phenotype changes [63, 64]. Further, the etiology of psoriasis could not be completely explained by TNF‐α signaling [65]. Thus, both LIGHT/HVEM/JNK/AP‐1 and TNF‐α signaling pathways contribute to the development and severity of psoriasis. Interestingly, AP‐1 is a pro‐inflammatory factor that directly regulates the expression of cytokines, including TNF‐α [66]. This means that AP‐1 could be a better therapeutic target than TNF‐α for the treatment of psoriasis. As mentioned previously, LIGHT is involved in bone development and promotes osteoclastogenesis. Selective AP‐1 inhibitors have been proven to prevent osteoclastogenesis in collagen‐induced arthritis, which is an autoimmune disease [67]. Thus, the LIGHT/AP‐1 signaling pathway plays a pivotal role in human autoimmune diseases and is potentially a highly efficient therapeutic target of such diseases.

While this study provides initial insights into the role of the LIGHT/HVEM pathway in psoriasis, several limitations should be considered when interpreting the results. The limited clinical cohort, while showing consistent qualitative results, lacked IHC quantification and correlation with PASI scores. Expanding the sample size with detailed clinical characterization remains an important direction for future validation. The experimental models primarily relied on immortalized keratinocytes, which, while valuable for mechanistic exploration, cannot fully replicate the complexity of primary human keratinocytes or the immune‐microenvironment in psoriatic skin. Furthermore, the findings await validation in animal models, which would provide important physiological context. Although key signaling mediators were identified, more comprehensive mechanistic profiling—such as transcriptomic or phospho‐proteomic analyses—was beyond the scope of this initial investigation. The observed crosstalk between LIGHT/HVEM and the IL‐23/Th17 axis, supported by bioinformatic analyses, will require further experimental validation in more physiologically relevant models. Finally, comparative studies with other TNF superfamily members and inflammatory skin diseases may help elucidate the specificity of the LIGHT/HVEM pathway in psoriasis. These aspects, while not addressed in the current study, provide a clear roadmap for future research.

## 6 Conclusion

We have demonstrated that blockade of HVEM and JNK/AP‐1 attenuates LIGHT‐induced increase in the viability and expression of IL‐6, IL‐8, PGI2, and PTGS2 in human keratinocytes. Our in vitro results suggest that the LIGHT/TNFSF14 axis may play a key role in regulating cytokine production by autoimmune cells and related pathways in psoriasis. Based on these findings, HVEM and JNK/AP‐1 warrant further investigation as potential therapeutic targets for psoriasis treatment.

## Author Contributions

**Cheng‐bin Ye:** data curation, writing – review and editing, formal analysis. **Cheng‐wen Fei:** data curation, project administration, writing– review and editing. **Ting Cao:** data curation, writing– original draft, supervision. **Xu‐yang Zhou:** validation, investigation. **Ying Zou:** conceptualization, methodology, supervision, resources, project. administration, funding acquisition, writing – review and editing.

## Ethics Statement

Approval was granted by the Ethical Committees of Shanghai Skin Disease Hospital prior to the data collection (No.2016‐12).

## Consent

All participants signed informed consent before the study.

## Conflicts of Interest

The authors declare no conflicts of interest.

## Data Availability

The data that support the findings of this study are available from the corresponding author upon reasonable request.
